# DNA Methylation Changes during *In Vitro* Propagation of Human Mesenchymal Stem Cells: Implications for Their Genomic Stability?

**DOI:** 10.1155/2013/192425

**Published:** 2013-10-30

**Authors:** Angela Bentivegna, Mariarosaria Miloso, Gabriele Riva, Dana Foudah, Valentina Butta, Leda Dalprà, Giovanni Tredici

**Affiliations:** Department of Surgery and Interdisciplinary Medicine, University of Milan-Bicocca, Via Cadore 48, 20900 Monza, Italy

## Abstract

Mesenchymal stem cells (MSCs) hold great promise for the treatment of numerous diseases. A major problem for MSC therapeutic use is represented by the very low amount of MSCs which can be isolated from different tissues; thus *ex vivo* expansion is indispensable. Long-term culture, however, is associated with extensive morphological and functional changes of MSCs. In addition, the concern that they may accumulate stochastic mutations which lead the risk of malignant transformation still remains. Overall, the genome of human MSCs (hMSCs) appears to be apparently stable throughout culture, though transient clonal aneuploidies have been detected. Particular attention should be given to the use of low-oxygen environment in order to increase the proliferative capacity of hMSCs, since data on the effect of hypoxic culture conditions on genomic stability are few and contradictory. Furthermore, specific and reproducible epigenetic changes were acquired by hMSCs during *ex vivo* expansion, which may be connected and trigger all the biological changes observed. In this review we address current issues on long-term culture of hMSCs with a 360-degree view, starting from the genomic profiles and back, looking for an epigenetic interpretation of their genetic stability.

## 1. Properties of Mesenchymal Stem Cells

Mesenchymal stem cells (MSCs) are multipotent adult stem cells with a great therapeutic potential in tissue engineering, regenerative medicine, autoimmune diseases, and pathologies characterized by chronic inflammatory processes [1, 2]. MSCs from bone marrow (BM-MSCs) are the best characterized adult stem cells but MSC-like populations have been isolated from several tissues such as adipose tissue, umbilical cord blood, skin, skeletal muscle, and also from dental tissues as dental pulp, exfoliated deciduous teeth, and periodontal ligament [3, 4]. Compared with other stem cell types, such as embryonic stem cells (ESCs) and neural stem cells, MSCs have several advantages and no ethical concerns limit their use. MSCs can be easily isolated, have a capacity for extensive proliferation and self-renewal, present a low risk of tumorigenicity, and can be used autologously. Moreover MSCs are considered immunoprivileged because they express low level of MHC-I molecules but not MHC-II and costimulatory molecules CD80, CD86, and CD40 [5]. The therapeutic effect of MSCs is mainly based on some key properties: (1) MSCs are able to differentiate not only into mesodermal lineages (osteogenic, adipogenic, and chondrogenic lineages) but also towards endodermal or ectodermal derivatives; (2) MSCs can exert strong anti-inflammatory and immunosuppressive effects; (3) MSCs can secrete many bioactive molecules affecting local cellular environment [6]. Finally, the capacity of MSCs to migrate preferentially to injured places, site of inflammation, and lymphoid organs allows different routes of administration [7].

A major problem for MSC therapeutic use is represented by the very low amount of MSCs which can be isolated from different tissues (e.g., in bone marrow the MSC population is 0.001–0.01% of the total cell number). To provide sufficient cell number for MSC clinical applications, after isolation an *in vitro* expansion phase is required. Differences in isolation methods, culture conditions, and seeding density greatly affect stem cell yield and properties [3, 8]. Different parameters are evaluated to optimize MSC expansion such as culture surface substrates, oxygen tension, medium composition, pH condition, and substitution of serum with plated-rich plasma [9, 10]. Furthermore the 3D expansion of MSCs on microcarriers could represent an interesting alternative to the conventional 2D monolayer culture method [9, 11]. Regardless of the culture conditions it is crucial that during the *in vitro* expansion MSCs retain their peculiar properties unchanged and no genetic alterations occur. 

## 2. hBM-MSCs: Really Stable at the Genomic Level?

Despite the clinical prospective of stem cell-based therapy, a few potential risks were recently described as the “risk profile” by Herberts et al. [12]. The hazard arises from the need of *in vitro* expansion and/or differentiation of human BM-MSCs (hBM-MSCs) before administration to a patient, and the malignant transformation is undoubtedly the more debated risk. In fact, the high proliferation rate in an artificial cell culture environment could favor the occurrence of genetic and epigenetic alterations. Since every cell division has a small chance of introducing deleterious mutations, it is generally known that chromosomal aberrations accumulate with age. In addition, numerous studies on tumour genotyping reported that genomic alteration is a hallmark of tumorigenesis [13, 14]. The main concerns are for autologous transplant applications in which the immune system is less efficient in eliminating potentially transformed cells. However, few publications reported spontaneous transformation of both adipose tissue and bone marrow-derived MSCs, after long-term *in vitro* culture expansion [15–17]. By contrast, other researchers supported the genomic stability of human MSCs (hMSCs) derived from different tissues [18–22]. On the other hand, genomic instability after long-term *in vitro* culture has been widely described in mouse and rat BM-MSCs [18, 19, 23–25], and it has been also associated with spontaneous malignant transformation [18, 19, 23, 24]. However, some reports on behalf of spontaneous transformation of hMSCs have been subsequently retracted from the same authors, since the results derived from contaminating tumor cell lines [26–28]. 

In this context, we had recently reported a general chromosomal stability of hBM-MSCs, though the occasional existence of transient clonal aneuploidies in two out of seven hMSCs samples [22]. In particular, in one case at least 52% of metaphases at passage 9 (P9) presented trisomy of chromosome 7; at P12 the same karyotype was found in 50% of the metaphases; moreover, in 11% of cells there was a loss of one chromosome X, so the total number of chromosomes was 46. In the second case, two equally represented subpopulations were evidenced at P4: a normal one, and a second with karyotype 49,XX,+5,+7,+9. However, for this sample, further analysis at later passages failed to reveal any clonal abnormalities, probably due to *in vitro *negative selection of the aneuploid clone. Moreover, a general stability of the genomic profile has been confirmed by array comparative genomic hybridization (a-CGH) analysis [22]. Similarly, Tarte et al. had revealed nonrandom aneuploidy in 5 of 20 hBM-MSC cultures, including recurring trisomy of chromosome 5 with occasional trisomy of chromosomes 8 and 20 [29]. Interestingly, 3 of 5 abnormal cultures were derived from the same donor, who provided two separate BM samples cultivated in either fetal calf serum and fibroblast growth factor or platelet lysate. These data suggest that recurring chromosomal alterations are not related to the specific culture conditions and could be donor-dependent. Once again, the abnormal karyotype did not persist on prolonged culturing demonstrating that all hBM-MSCs, with or without chromosomal alterations, showed progressive growth arrest and entered senescence without evidence of transformation either *in vitro* [22] or even *in vivo* [29]. Also Binato et al. demonstrated chromosome variability after passage 4 in nine cultures of hBM-MSCs using conventional cytogenetic analysis [30]. They showed that seven of the nine cultures presented random aneuploidy, but the abnormalities were lost by the next passage. Nevertheless, in one culture, a clonal abnormality was identified from passage 6 to passage 8. However, at the molecular level, changes were observed from passage 5 onwards, indicating initiation of differentiation, reduction in proliferation, and potential induction of senescence in all analysed samples, including even those with karyotypic abnormalities. Therefore, these genetic alterations are not associated with a selective growth advantage *in vitro*; indeed they conferred a growth disadvantage to abnormal cells, probably linked to DNA damage-associated senescence [31] or through a not yet well-defined internal mechanism of self-regulation. 

In the literature a link between ageing/senescence and genomic stability is often reported [32], as well as between hypoxia and ageing/senescence [33], due to a compromised DNA repair gene activity. Furthermore, experimental data have indicated that hypoxia causes downregulation of DNA mismatch repair (MMR) genes and genomic instability in stem cells via specific epigenetic events [34]. For these reasons, we should not neglect the effects of hypoxia on long-term culture of hMSCs, although it is not the central focus of this review, addressing the topic on genomic stability. Whereas many authors agree that hypoxia enhances proliferation, inhibits senescence, and maintains stem cell properties of hMSCs [35–38], data on the effect of hypoxic culture conditions on genomic stability are few and contradictory. Some authors argue that hypoxic hMSCs maintains normal chromosome karyotype and intact genetic integrity [37], and others argue the exact opposite [39] claiming that amplification of hMSCs in a low-oxygen environment facilitated chromosomal instability via repeated cell division. In addition, a high frequency of detected chromosomal abnormality breakpoints corresponded to common fragile sites (CFSs), in analogy with tumorigenesis [40, 41]. Considering these conflicting data, the question is still open and particular attention should be given to the use of low-oxygen environment, through continuous monitoring of the chromosomal stability in addition to the proliferative capacity and differentiation of hMSCs. Finally, it should be remembered that the impact of culture condition on epigenetic properties of pluripotent stem cells and preimplantation embryos, for example, has already been established [42].

## 3. How Measure Cell Ageing?

As anticipated in the previous section, culture expansion of hBM-MSCs is limited and after a certain number of cell divisions they enter a senescent state and ultimately stop proliferating. This phenomenon, the “Hayflick limit” [43], also known as replicative senescence, restricts the life span *in vitro* of all primary mammalian somatic cells. Senescent cells are mitotically arrested, and thus they are not dead and remain metabolically active. In this condition, the majority of cells acquire a characteristic large and flat fried egg morphology [22]. Since the first discovery of the “Hayflick limit” several studies have shown an inverse relationship between donor age and the replicative life span *in vitro* for MSCs [44, 45], proving that the age of an organism can have an influence on MSC proliferation. However, there is a high variation between different donor samples [22, 46].

It is hard to predict at which passage or number of cell divisions MSCs are approaching replicative senescence. First of all it would be necessary to identify a standardized system to track long-term culture [47]. Although many groups provide the number of passages as an indicator for cellular ageing, this approach is largely dependent on number of cells that have been seeded as well as confluence at the time of harvesting [48]. Population doublings (PDs) may provide a more accurate measure for cellular ageing and were calculated as quotient of the number of cells harvested divided by the number of cells that have been initially seeded [49]. However PDs do not include apoptosis or necrosis, which affect cell number. Nevertheless, despite standardized culture methods, there is considerable variation between different donor samples [48]. Even if the formulae can be modified to take into account the cell culture time, the population doubling time (PDT) still has the same limits [22]. So far, the only well-established method to quantify the amount of senescent cells is the senescence-associated *β*-galactosidase (SA-*β*-gal) staining [50]. Although this enzyme is active only in senescent hMSCs, unfortunately this staining does not facilitate absolute quantification of the senescent state. 

In conclusion, there is no golden standard in the measurement of cell ageing and a more specific molecular marker would be necessary in order to grade the level of senescence of hMSC preparations.

## 4. Telomere Length and Differentiation Ability: Two Sides of the Same Coin

Telomeres consist of a repeated sequence located at each end of each chromosome. This repeated sequence is required for chromosomal stability and integrity, functions closely connected with for both cancer and ageing [51]. It has been proposed that the progressive shortening of the telomeres is the main trigger for replicative senescence, because it functions as an internal clock and the number of telomere repeats decreases at every cell division [48]. However, it is still being debated whether telomere shortening is really the initiating mechanism or whether it is instead an effect of replicative senescence [52–54]. Telomeric loss results in a variety of consequences such as inhibition of mitosis, genotoxic damage due to accumulation of free radicals, and chromosomal rearrangement, which may trigger a DNA damage response leading to senescence and cell apoptosis [55, 56]. The length of telomeric ends is controlled by telomerase, a ribonucleoprotein complex whose RNA and protein components were both essential for activity [57]. Telomerase is constituted by a catalytic unit with reverse-transcriptase activity (Tert) and RNA component (Terc) that serves as template for telomere extension [58]. Pluripotent cells, such as germ line cells, embryonic stem cells, and induced pluripotent stem cells, can bypass the barrier of senescence by telomerase expression. On the contrary, telomerase activity and hTERT transcripts were not expressed in cultured MSCs [20, 59], and progressive shortening of the telomeres has been demonstrated during *ex vivo* expansion of MSCs derived from human and non-human primate [22, 60–62]. On the other hand, the transformed hBM-MSCs described by Wang et al. exhibited telomerase activity [16]. Even though cancer cells have been shown to have increased levels of telomerase activity [63], constitutive expression of TERT by itself does not generate malignant conditions as it does not cause growth deregulation [64]. Thus, the barrier of senescence may be advantageous for hMSCs since it reduces the risk of oncogenic transformation upon prolonged *in vitro* culture [65]. On the other hand, the senescence may be disadvantageous for hMSCs since it may impair their differentiation capability. Indeed, long-term culture has a significant impact on differentiation capacity of hMSCs, especially towards adipogenic lineage [46, 60, 66–68].

It has been recently demonstrated that ectopic expression of telomerase can immortalize hMSCs maintaining the differentiation potential *in vitro* toward the osteoblastic and adipogenic lineages [69, 70]. The generation of a hMSC line expressing TERT that exhibits enhanced cell proliferation and stability in cell culture could be a new strategy for both basic and applied tissue engineering studies of bone development and repair [70]. Finally, the progressive shortening of the telomeres seems to be a self-regulating mechanism able to reduce the risk of oncogenic transformation of MSCs in culture, since it can limit expansion of potentially malignant cells.

## 5. Epigenetic Program and Differentiation Potential in hMSCs

Gene expression potential in stem cell renewal and differentiation is regulated by epigenetic mechanisms that alter the transcriptional permissiveness of chromatin, of which DNA methylation (DNAm) is the best characterized component [71]. DNAm consists in the addition of a methyl group to the carbon 5 of the cytosine into CpG contexts and it is involved in development and cellular differentiation [72]. However, DNAm does not work alone, since histone modifications and noncoding RNA regulation collaborate in controlling chromatin plasticity. It is commonly accepted that DNAm silences gene expression. Actually, gene expression depends on promoter CpG content, with methylated high-CpG content promoters being usually inactive, while methylated low-CpG content promoters can be active or inactive [73]. Thus, the “open” chromatin, that is, “global DNA hypomethylation” and abundance of transcriptionally active chromatin marks, such as trimethylated H3K4 (H3K4me3) and acetylation of histone H4, correlates with the ability to activate a wide range of cell type-specific genes during the differentiation programs [71]. The maintenance of the pluripotency state in ESCs is given by development-associated transcription factors, such as *OCT4*, *NANOG*, and *SOX2*, which activate genes of self-renewal at their unmethylated promoters [74]. Differentiation of ESCs is due to methylation of these pluripotency genes such as *OCT4*, determining their downregulation [75]. MSC epigenetic profiles reflect a more limited differentiation potential as compared to ESCs (that is why MSCs are better classified as multipotent than pluripotent), but numerous epigenetic modifications occur concomitantly during both osteogenic and adipogenic differentiation [76]. In adipose tissue stem cells (ASCs) and BM-MSCs *OCT4* is silenced by promoter hypermethylation, whereas *NANOG *and *SOX2* are unmethylated despite the repressed state of the genes [77], indicating the implications of other chromatin-based mechanisms, such as post-translational histone modifications. Epigenetic studies from the laboratory of Collas suggested a model of epigenetic commitment or preprogramming of MSCs toward particular lineages. They affirmed that post-translational histone modifications on promoters contribute to establishing a permissive state of differentiation but cannot predict transcriptional activation outcome [78]. In this issue several studies evidenced the role of histone H3K9Ac and H3K9Me2 modifications (associated to gene activation and gene silencing, resp.) in regulation of MSC fate commitment and ultimately predict cell fate. Tan et al. identified several differentially expressed genes regulated by acetylation of H3K9 (H3K9Ac) and/or dimethylation of H3K9 (H3K9Me2), implicating their role in hMSC osteogenic differentiation [79]. Similarly, Li et al. showed the role of histone H3 acetylation in regulating MSC ageing and spontaneous osteogenic differentiation [80]. Interestingly, they demonstrated that the basic fibroblast growth factor (bFGF) promoted MSC proliferation and suppressed its spontaneous osteogenic differentiation, modulating histone H3 acetylation in the *OCT4* gene. Very recently, Wang et al. showed that low concentrations of trichostatin A (TSA), a histone deacetylase inhibitor, prevented the spontaneous differentiation of human umbilical cord MSCs during long-term culturing, delaying their ageing [81]. In conclusion, the crucial role of post-translational histone modifications in regulating the differentiation potential of MSCs provides a system for their selective manipulation in order to hinder their ageing *in vitro*. 

## 6. Epigenomic Modifications during hBM-MSCs *In Vitro* Expansion: Random Fluctuations or Thin Autoregulation? 

More specific studies have recently addressed the relationship between epigenetic changes acquired during culture of MSCs and their functional changes. Wagner et al. speculated that replicative senescence and ageing might be regulated by similar mechanisms [82]. DNAm patterns were overlapping and maintained throughout both long-term culture and ageing, and highly significant differences were observed only at specific CpG sites, associated with promoter regions, especially in homeobox genes and genes involved in cell differentiation [83]. In this context, the group of Wagner defined the “Epigenetic-Senescence-Signature” as the senescence-associated DNAm (SA-DNAm) changes, which were related to age-associated modifications in MSCs from young versus elderly donors and could be used to monitor senescence for quality control [84]. Schellenberg et al. [85], analyzing functional, genetic, and epigenetic sequels of long-term culture of hMSCs demonstrated that the DNAm profiles differed markedly in MSCs from adipose and bone marrow, also confirming the data on gene expression profiles of other studies [86, 87]. Furthermore, Schellenberg et al. evidenced that senescence-associated hypermethylation and hypomethylation were often localized to regions with repressive histone marks, such as abundance of H3K9me3, H3K27me3, and targets of the histone methyltransferase EZH2 [85]. Interestingly, EZH2, a component of the polycomb-repressive complex 2 (PRC2), has previously been implicated in replicative senescence: its levels are downregulated in senescent cells, so the program of differentiation is permitted [88]. On the contrary, EZH2 overexpression is associated with several cancers [89, 90]. Furthermore, it was found that genes which are targets of the polycomb group proteins (PCG) undergo hypermethylation with age, hindering cell differentiation [91]. Thus, age may contribute to carcinogenesis by irreversibly silencing genes that are suppressed in stem cells and by stabilizing stem cell features. 

Two recent studies compared the methylation profiles during *in vitro* expansion of MSCs. Choi et al. [92], comparing differential methylation patterns between early and late passages of hBM-MSCs, evidenced that hypermethylation increases at genes related to DNA replication, cell cycle, and adipogenic differentiation, due to long-term culture. 

**Figure 1:**
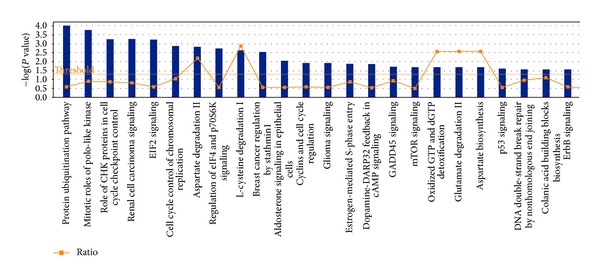
This graph shows the most statistically significant pathways involved in methylation variations of gene promoters between early and late passages of hBM-MSCs, obtained by Ingenuity Pathway Analysis (IPA). Blue bars indicate −log(*P* value), while the orange squares indicate the ratio of input list genes that map to the considered pathway divided by the total number of genes involved in this specific pathway.

In our study [22] we performed a gene ontology (GO) analysis on genes with a change in the methylation status from early to late passages in hBM-MSCs. We identified several correlations between the functional changes and the change of methylation profiles of hBM-MSCs both acquired during culture. As an example, the categories “cell signaling” and “apoptosis and cell death”, including genes that have essential functions for the viability and functionality of MSCs, were unmethylated at early passages and remained so even at late passages; thus, they should not be turned off. Among the “methylated gene promoters”, which could be inactivated with increasing passages, we found several metabolic processes such as genes for lipid and fatty acid metabolic process. These data well correlate with the decreased adipogenic differentiation potential during long-term culture. Furthermore, we analyzed our DNA methylation data by Ingenuity Pathway Analysis (IPA). IPA software examines functional relationship within an input list of genes and identified which pathways, from the IPA library of canonical pathways, are most significantly associated to the data set [93]. In our study, the input list was represented by genes whose promoters modify their methylation status between early and late passages (unpublished data). The most statistically significant pathways involved in methylation variations of gene promoters between early and late passages of culture are shown in Figure 1, and among these numerous concern cell cycle regulation, DNA repair, metabolism, and cancer.

## 7. Functional Consequences in hMSCs during Long Term-Culture: Everything Changes around, but an Apparent Genomic Stability Remains

Long-term *in vitro* expansion alters the biology of adult MSCs and induces tightly regulated epigenetic modifications. However, the genome of hMSCs appears to be relatively stable and so far malignant transformation upon hMSC transplantation has not been observed in clinical trials [29]. One might speculate that the genomic stability is somehow guaranteed in hMSCs during *in vitro* long-term culture. As mentioned above, the abnormal karyotype generally did not persist on prolonged culturing, probably due to DNA damage-associated senescence. Epigenetic changes might therefore antagonize some genetic alterations arisen during long-term culture of hMSCs.

**Figure 2:**
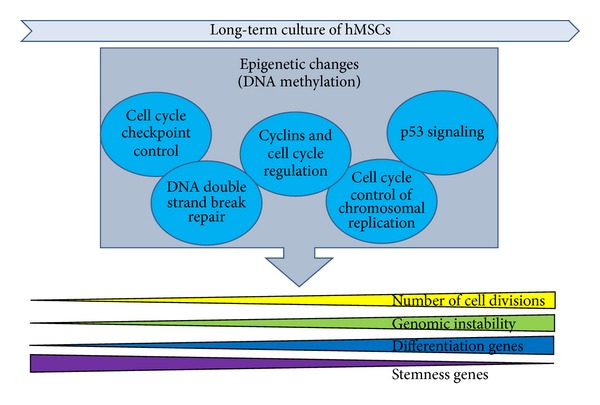
Epigenetic changes (in particular DNA methylation) at specific gene promoters during long-term culture of hMSCs may regulate processes like senescence and proliferation but also genomic stability. Many statistically significant pathways involved in methylation variations between early and late passages of Figure 1 concern cell cycle regulation, DNA repair, and cancer (light-blue circles).

Izadpanah et al. have provided data supporting this hypothesis, analyzing the transcriptome of both ASCs and BM-MSCs, at early and late passages, in human and rhesus macaque [94]. All MSCs have altered cell cycle progression, resulting in both cellular crisis and senescence. In addition, hMSCs underwent an increase in the frequency of cells in the S phase at P20 and higher. However, extended culture of hMSCs failed to reveal any chromosomal alterations, whereas all rhesus MSCs (rMSCs) displayed an aneuploidy karyotype. Gene ontology analysis indicated that genes involved in protein metabolism, protein catabolism, and regulation of pol II transcription were over-represented in rASCs, whereas those involved in the regulation of cell cycle and regulation of I*κ*B/nuclear factor-*κ*B (NF*κ*B) cascade were over-represented in hBM-MSCs. These data showed a correlation between the observed differences in karyotype changes and gene expression changes between rMSCs and hMSCs. Thus, hMSCs during *in vitro* expansion could trigger a specific program in order to protect the integrity of the genome, preventing genetic instability via arrest in S phase and involving p53 and NF*κ*B pathways, both expressed in hBM-MSCs but not in rASCs [94]. Similarly, our IPA analysis of methylation changes of gene promoters between early and late passages of hBM-MSC cultures evidenced the implication of several anticancer pathways, suggesting that the genomic stability observed in hBM-MSCs during long-term culture may be determined by the methylation changes at specific gene promoters (Figure 2). 

## 8. Conclusion

The intimate correlation between DNA methylation, stem cell renewal and differentiation and between stem cell culture condition, genomic instability, and cell proliferation is now evident. The study of the mechanisms for the genomic integrity maintenance could be useful not only for standardization and safety of hMSCs for therapeutic applications but also for cancer prevention, risk prediction, detection, prognosis, and therapy.
